# A GIS-based method for revealing the transversal continuum of natural landscapes in the coastal zone

**DOI:** 10.1016/j.mex.2018.05.012

**Published:** 2018-05-21

**Authors:** Artan Hysa, Fatma Aycim Turer Baskaya

**Affiliations:** a Graduate School of Science, Engineering, and Technology, Istanbul Technical University, ITU Ayazaga Kampusu, 34496, Maslak, Istanbul, Turkey; b Department of Landscape Architecture, Faculty of Architecture, Istanbul Technical University, Harbiye Mahallesi, Taskisla Cad., 34367, Sisli, Istanbul, Turkey

**Keywords:** ModelBuilder, Transversal Continuity Depth (TCD), ModelBuilder, ArcGIS, CORINE Land Cover, ICZM, Sustainable Coastal Tourism

## Abstract

The method presented in this article is helpful for analyzing the landscape properties and unfolding the transversal continuity of natural landscapes in the coastal zone. The novel conceptual approach to analyze the landscape structure in the transversal direction with reference to coastline is different from others focusing on the longitudinal analysis of landscape properties in the coastal areas. The procedure is relying on the fundamental questioning of the spatial relation of each landscape patch with the coastline. The raw material is Land-Use/ Land-Cover (LULC) data. At this stage the method is tested successfully utilizing CORINE Land Cover (CLC) data. The method is structured in four sequential stages, and formalized via ModelBuilder/ ArcGIS software into a model applicable to any coastal zone. The output of each phase is used as the raw material of the following stage. The presented method is useful in identifying a set of endangered natural landscape patches located as a hinge in between two transversally connected natural landscape mosaics (TCNLM). A second set is highlighted as potential artificial surfaces located as barriers between the coastline and TCNLM. The presented method is useful in the analysis stages of Integrated Coastal Zone Management (ICZM) and Sustainable Coastal Tourism (SCT).

• *The presented procedure focuses on the transversal landscape structure in the coastal zone rather that the classical longitudinal analysis of coastal landscapes.*
• *The procedure brings a new way of CORINE Land Cover data utilization beyond its basic monitoring objective, useful for a variety of decision making and management processes such as; Integrated Coastal Zone Management (ICZM), Sustainable Coastal Tourism (SCT), Environmental protection, Landscape connectivity, etc.*
• *The method builds a novel tool set customized via ModelBuilder in ArcGIS, being applicable to any coastal zone.*

## Graphical abstract

**Figure:**
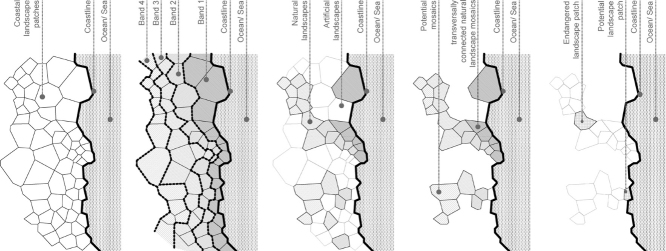

**Specifications Table**

**Table:** 

Subject area	• *Earth and Planetary Sciences*
More specific subject area	*Coastal Zone Management, Environmental Protection, Landscape Connectivity*
Method name	*ModelBuilder, Transversal Continuity Depth (TCD)*
Name and reference of original method	*Hysa, A., & Türer Başkaya, F. A. (2018). Revealing the Transversal Continuum of Natural Landscapes in Coastal Zones- Case of the Turkish Mediterranean Coast. Ocean & Coastal Management, 158, 103-115.*
Resource availability	*CLC data source:*http://rod.eionet.europa.eu/obligations/572/deliveries *ModelBuilder Diagrams are shared with this article.*

## Method details

### Conceptual approach: the concept of band

**Fig. 1:**
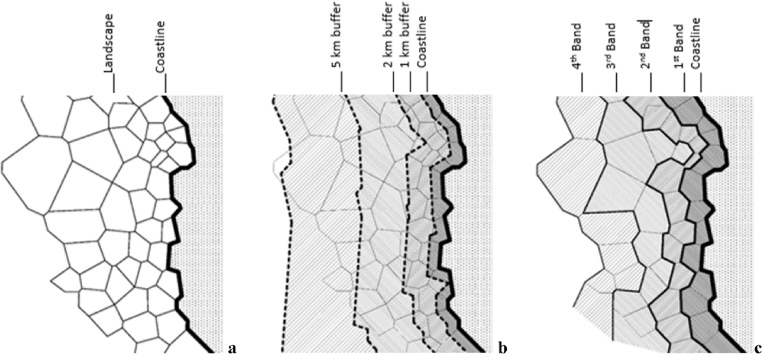
The comparison between the fixed buffer zone approach (b) and the concept of bands (c) in coastal landscapes (a).

The method presented in this article originates from an unusual conceptual approach to the analysis of landscapes in the coastal zone (Fig. 1a) as developed in our previous research [1]. Generally, the coastal landscapes are investigated in their longitudinal structure along the coastline, leading to the widely used approach of fixed buffer strips (Fig. 1b) [2]. In contrast, our approach is focusing on the transversal formation of landscapes along the coastal zone. Investigating the landscape structure from the coastline further inland, leads to the novel *concept of bands* (Fig. 1c). More precisely the band level refers to the adjacency order a certain landscape patch has with the coastline considering the later as the initial spatial reference. This new approach is profoundly settled on the organic structure of landscape patches in the territory (Fig. 1c), much different from the fixed buffer strips being an inorganic zoning (Fig. 1b).

adapted from [1]

The procedure is formalized into a model/ toolset via ModelBuilder in ArcGIS 10.2.2 software. ModelBuilder is accepted as a visual programming language useful in constructing reprocessing workflows in the form of models. The formalized models consist of stringed sequences of geo-processing tools by providing the output of the previous operation as the input of the next one [3]. The usage purposes of Model Builder is of a very wide range but in this experiment it can be considered to help in developing a model as a customized tool unique to the goals of the study [4].

**Fig. 2:**
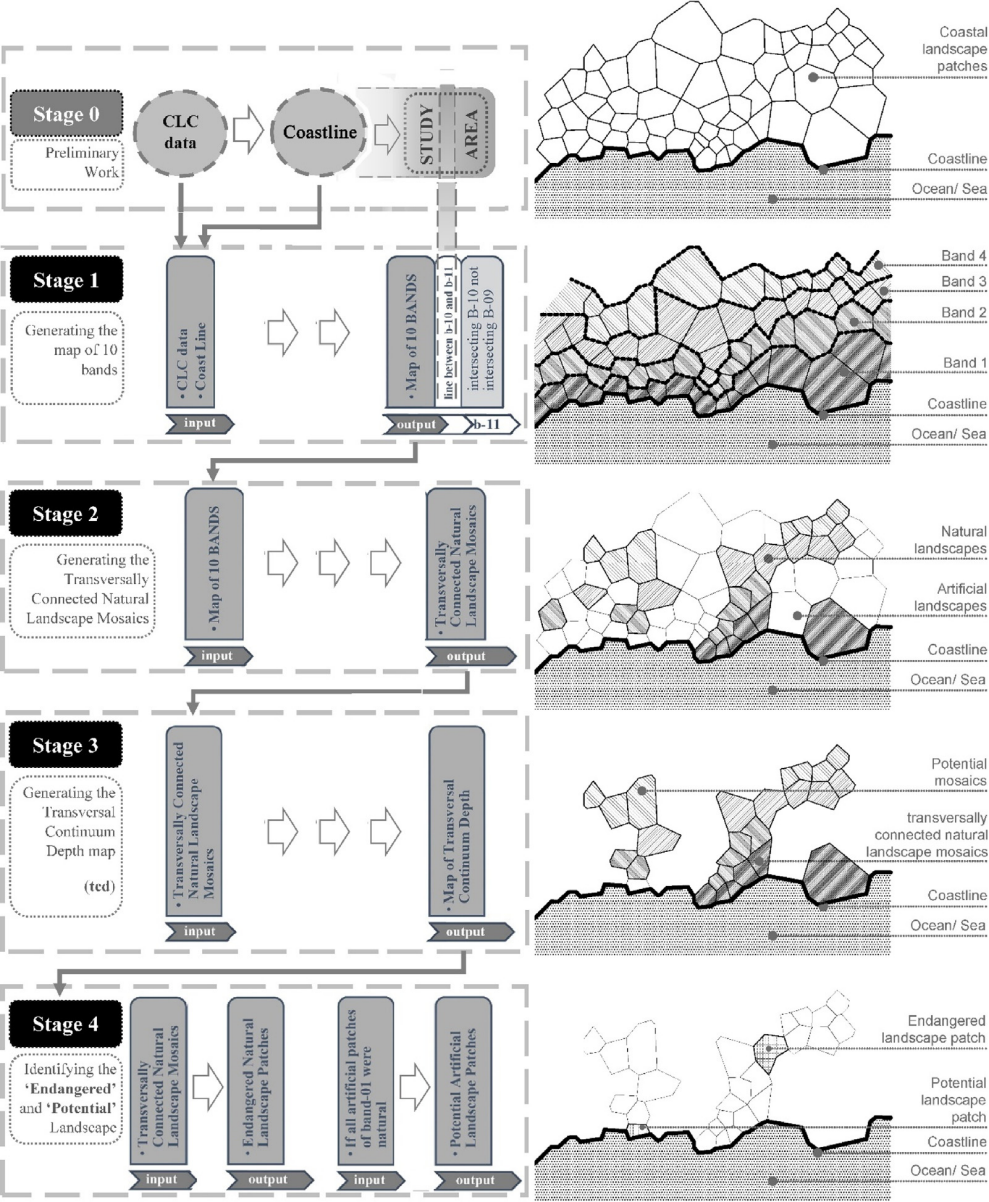
The workflow of the method and the conceptual graphics per each stage.

The workflow consists of one preparatory and four analytical stages (Fig. 2). First, the input parameters of the process, being the land cover data and the coastline feature, are derived from Corine Land Cover (hereafter CLC) geospatial data as the raw material of this study. Both inputs are introduced into the process of the Stage 1 generating the set of 10 bands (Fig. 2). The output of the Stage 1 is the main input of the Stage 2 which results in the set of *transversally connected natural landscape mosaics* (hereafter TCNLM). The main goal of the Stage 3 is the reclassification of each landscape patch of TCNLM by the maximum band level they provide transversal connectivity for, by assigning the *transversal continuity depth* (hereafter TCD) value. Last, at the Stage 4 there are defined a set of endangered natural landscape patches and a further set of potential artificial land cover surfaces.

The endangered natural landscapes set consists of patches which are located as singular hinges in-between two TCNLM. Generally, these patches belong to middle bands such as, the band 5, the band 6, or the band 7. Their endangered status relies on the assumption that if such unique natural landscape patches were artificialized, they would cause landscape fragmentation to TCNLM in the coastal zone. Similarly, the set of potential artificial landscape units includes artificial patches belonging to the band 1 (located in the waterfront), which act as barriers between the coastline (sea/ ocean) and the TCNLM. These artificial patches are labeled as potential based on the assumption that if they were recovered/ restored close to their natural state, they would provide extensive connectivity between the coastline and TCNLM.

### Stage 1 in detail

**Fig. 3:**
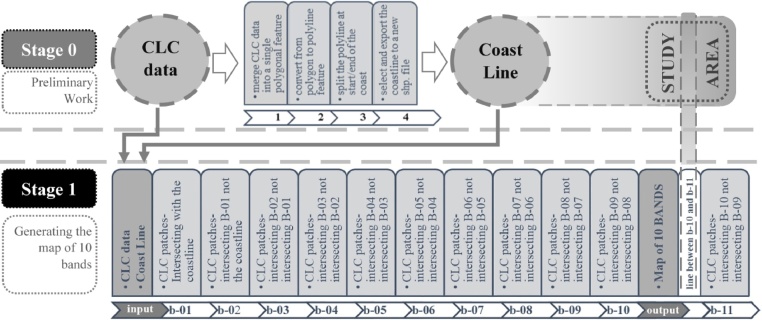
The detailed sequences of the preparatory Stage 0 and the Stage 1 of the workflow.

**Fig. 4:**
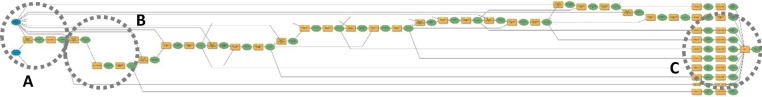
The full workflow sequences of the Stage 1 as modeled in ModelBuilder.

The Stage 1 of the procedure is preceded by a preliminary stage (Stage 0 in Fig. 2, Fig. 3) aiming the derivation of the geospatial polyline feature of the coastline. First, CLC data in the coastal zone is merged into a single polygon shapefile which further is converted into a polyline geometry. The polyline feature is split at the start and ending points of the coastline generating the geospatial shapefile of the coastline being the second input data for the Stage 1. The main goal of the Stage 1 is to reclassify all CLC patches by their adjacency order in spatial relation with the coastline. As a result, each land cover patch is assigned a new unique value of *band level*. The workflow of the Stage 1 is formalized via ModelBuilder (Fig.4).

adapted from [1]

Referring to Fig. 4 and Table 1, the workflow of the Stage 1 consists of three core subsequences. In the first subsequence A, the raw CLC data is tested for the adjacency with the coastline. For this, coastline and CLC data were defined as parameters (S1-a1). Then, the selected CLC patches in touch with the coastline (S1-a2) are exported as the patches of the band 1 (S1-a3). In the second subsequence B, the raw CLC data is re-tested for the adjacency with the formerly defined patches of the band 1 (S1-b1). The selected patches excluding the band 1 patches (S1-b2) are categorized as the band 2 (S1-b3). The subsequence B is repeated for defining all further bands. Finally, the set of patches of each band is added a new attribute value (S1-c1) of band level (S1-c2), and further merged (S1-c3) into a single geospatial file (.shp) to be utilized as the input in the Stage 2.

**Table 1:** Key ModelBuilder sub-sequences of the workflow of the Stage 1.

### Stage 2 in detail

**Fig. 5:**
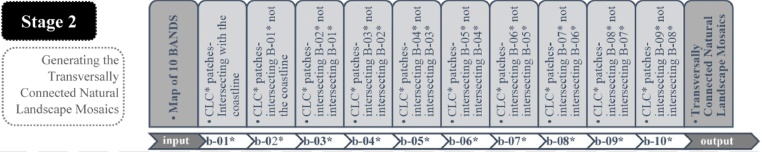
The detailed sequences of the Stage 2 of the workflow.

The map of 10 bands derived as the output of the Stage 1 is the raw material (input) for the Stage 2 (S2-a1) aiming to reveal the TCNLM along the coastal zone. The procedure of the Stage 2 initiates with a filtering operation of natural CLC patches of each band (S2-a3). The natural surfaces consist of the following CLC classes; clc-523, clc-522, clc-521, clc-422, clc-421, clc-411, clc-333, clc-331, clc-324, clc-323, clc-321, clc-313, clc-312, clc-311. In other words, the artificial surfaces (clc-100) and the agricultural areas (clc-200) are excluded at this stage (Fig. 5).

adapted from [1]

The set of natural land cover classes may tolerate some exceptions due to the specifics of the context of the study area. For example, in the Mediterranean context the olive groves (clc-223) and agro-forestry (clc-244) areas are considered part of the natural environment [5,6], and are included as shown in the case of Turkish Mediterranean coastal zone by Hysa & Türer Başkaya [1]. The process of the Stage 2 runs similar to the Stage 1, searching for natural landscape patches within each band, that is sharing borders with any natural land cover patch belonging to the preceding band.

**Fig. 6:**
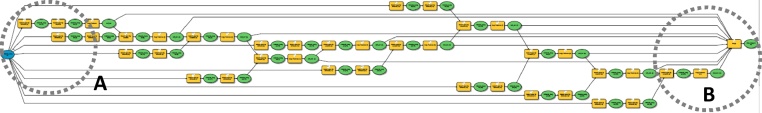
The full workflow sequences of the Stage 2 as modeled in ModelBuilder.

As shown in the full workflow of the Stage 2 in Fig. 6 and further in detail in Table 2, the sequence starts with setting the 10 band data as the main parameter (input) of the process (S2-a1). First, *selecting by attributes* there are filtered the patches of the band 1 (S2-a2). A further filter is performed aiming the exclusion of artificial and agricultural surfaces (S2-a3). The remaining natural landscape surfaces are exported as the set of natural surfaces in the frontline of the coast (S2-b2). The sub-sequence (S2-A) is run again for the band 2. The natural landscape patches of the band 2 that intersects with the natural surfaces of the band 1 (S2-b1) are exported as transversally connected natural landscapes of the band 2 (S2-b2). The remaining natural surfaces of the band 2 that are not connected with natural surfaces of the band 1 are excluded from the further steps of the workflow. The same sub-sequence repeats for all remaining bands resulting in a set of natural landscape patches that are interconnected. The merged data consist of the TCNLM along the coastal zone.

**Table 2:** Key ModelBuilder sub-sequences of the workflow of the Stage 2.

### Stage 3 in detail

The Stage 3 consists of a reclassification procedure for each patch of the TCNLM as derived from the Stage 2. The patches are classified by their maximum band level they provide transversal connectivity for. For example, a patch belonging to the band 2 and being part of a TCNLM that stretches further inland to the band 8, is assigned a TCD value of 8. In this context, the patches having a low band level (1, 2, 3, 4) and a high TCD value (7, 8, 9, 10) can be considered crucial components of TCNLM structure. In other words, those natural landscape patches are closer to the sea but providing deeper natural landscape continuity further inland.

**Fig. 7:**
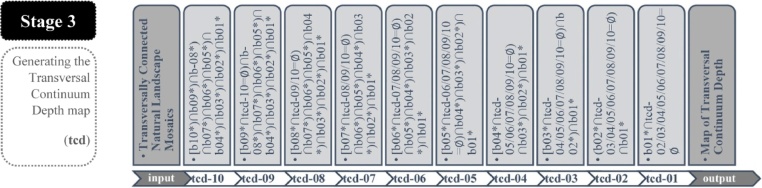
The detailed sequences of the Stage 3 of the workflow.

**Fig. 8:**
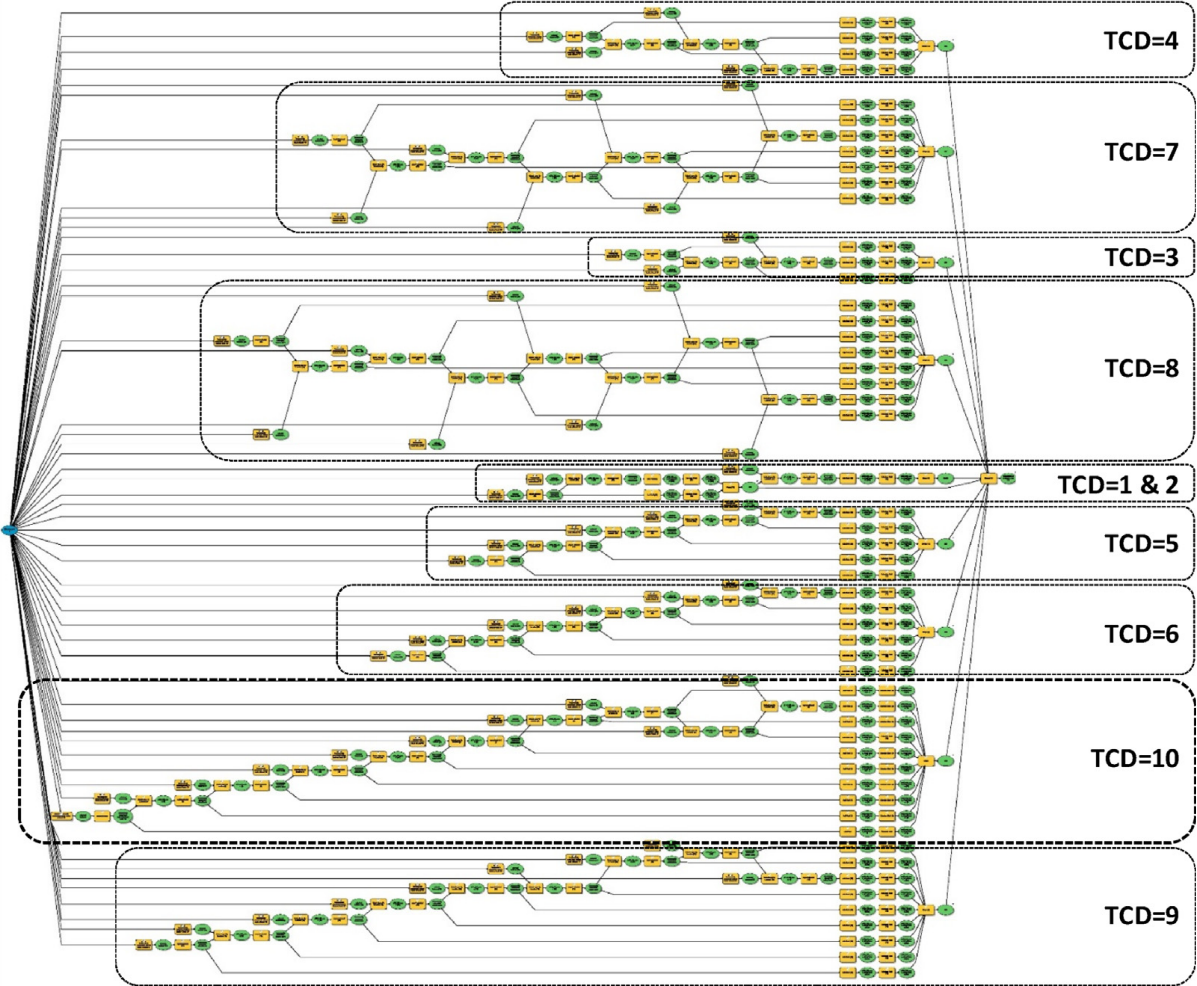
The full workflow for classifying TCNLM patches by their TCD values structured in ModelBuilder.

The workflow of the Stage 3 as represented in Fig. 7 is flowing in the reverse order compared with the flow of the Stage 1 and the Stage 2. It tests the transversal adjacency condition staring from the band 10 rather than the coastline. The aim here is to assign to each TCNLM patches the maximum band level further inland they provide connectivity for. Referring to Fig. 7 the first step is the most complex one, checking for interconnection among all bands in a descending order. In each consecutive step the string becomes shorter due to the reduction of bands under investigation. The visual information in Fig. 8 is in the same line with this fact. The full ModelBuilder workflow sequences in Fig. 8 are representing the complexity of each sequential string, resulting in one merged geospatial data of TCNLM reclassified by their TCD values.

adapted from [1]

First, the reclassification initiates by setting TCD value of 10 to all patches of band 10 (hereafter b10t10). Next, TCD value of 10 is assigned to the patches of the band 9 that are adjacent with b10t10 resulting in b9t10. Similarly, TCD value of 10 is assigned to all patches of further lower bands that are transversally connected to the preceding ones in descending order. After finalizing the sequential string defining TCD = 10, in the next step the reclassification operates on the un-classified patches only. Thus, TCD value of 9 is initially assigned to all patches of the band 9 that have not been assigned a TCD value of 10 in the preceding sequence. Similarly, the procedure continues with the following bands in descending order as in the first sequence. The same logic is repeated for the remaining TCD values going less in complexity. Finally, TCD value of 1 is assigned to natural landscape patches that are adjacent to the coastline only and are not transversally connected to any patch of the band 2.

**Fig. 9:**
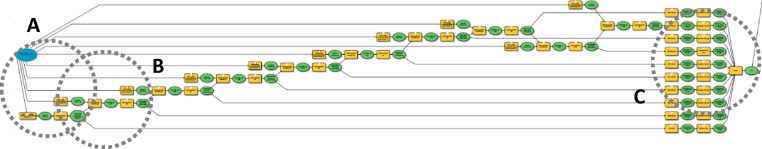
The workflow sequences of the Stage 3 as modeled in ModelBuilder for assigning TCD value of 10.

Further in detail, Fig. 9 and Table 3 brings a closer look to the sequential string of TCD = 10. First, the TCNLM data is set as the main parameter (S3-a1). Then, the patches of the band 10 are selected based on their attributes (S3-a2), and further are exported (S3-a3) as patches to be assigned a new value (S3-c1) of TCD = 10 (S3-c2). The sequence continue by filtering TCNLM data highlighting patches of the band 9 (S3-b1) not assigned any TCD value yet (S3-b2). The defined patches (S3-b3) are assigned the TCD value of 9.

**Table 3:** Key ModelBuilder sub-sequences of the workflow of the Stage 3 for assigning TCD value of 10.

The process runs the same with the following bands in a descending order, resulting in the merged set of patches belonging to various bands but sharing a TCD value of 10 (S3-c3). Regarding the reclassification of the remaining TCD values the process runs in a similar way according to the procedure explained in Fig. 7, Fig. 8.

### Stage 4 in detail

**Fig. 10:**
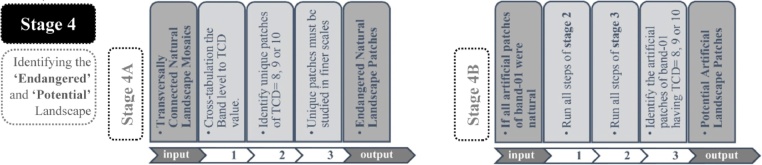
The sequences of the Stage 4 A and the Stage 4B of the workflow.

The presented three stages consist of data analysis procedures based on the existing attributes of CLC datasets. Whereas the Stage 4 stands on two interpretive evaluation of the results of the previous stages (Fig. 10).

adapted from [1]

For example, the Stage 4A relies on the cross-tabulation of band level to TCD value for each TCNLM patch (Table 4). As previously stated, the patches attributed by lower band level and high TCD value can be considered as crucial components of TCNLM. Moreover, if these patches are unique within the TCNLM they belong to, they can be highlighted as engendered natural landscape patches as illustrated in previous studies [1].

**Table 4:** The cross-tabulation of band level to TCD value for each TCNLM patch.

A second interpretive analysis relies on the assumption if all artificial patches belonging to the band 1 were natural landscape surfaces. The assumption is introduced in the process at the initial steps of the Stage 2. During the filtering process (S2-a3), there are filtered the artificial surfaces instead of the natural ones. The rest of the process runs identical with the remaining workflow sequences of the Stage 2 to identify TCNLM that connects to the coastline through an artificial land cover patch. The aim of the Stage 4B is to identify artificial patches belonging to the band 1 that separate the coastline and TCNLM which starts at the band 2. The highlighted artificial surfaces if restored/ reclaimed can provide extensive connectivity to TCNLM to the coastline.

The method and procedures presented in this article are useful for several research fields sharing the coastal zone as the common study area, as discussed in our initial study [1]. For example, the method can be of use within the Sustainable Coastal Tourism (hereafter SCT) agendas. One of the main goals of SCT is to minimize the impact of the overloaded touristic infrastructure on the coastline by advocating a transversal spatial spread of the touristic interest providing diversified touristic activities along a spatial gradient from the coastal to mountainous lands [7]. At the same time, the goal to widen the spatial scope of coastal zone studies is present within the core objectives of Integrated Coastal Zone Management (ICZM). One of the seven principles of ICZM urges for the inclusion the offshore and upland environments (ocean-land interlinks) in coastal zone studies, as part of the wider geographical context [8]. The emphasis on the assessment of natural landscape connectivity by the presented method implicates it with ecological studies which aim to assess and preserve the natural landscapes as crucial components of environmental protection and sustainable development.
